# Supplementary material for: Pigments to precision: RUBY aiding genetic transformation and genome editing in wheat and barley
Source: Physiol Mol Biol Plants. 2025 May 15;31(4):545–54. doi: 10.1007/s12298-025-01591-5 (PMC12116407; doi:10.1007/s12298-025-01591-5)
Supplement: Supplementary file 1 — Supplementary file1 (PPTX 31117 KB) [file 12298_2025_1591_MOESM1_ESM.pptx]

## Slide 1
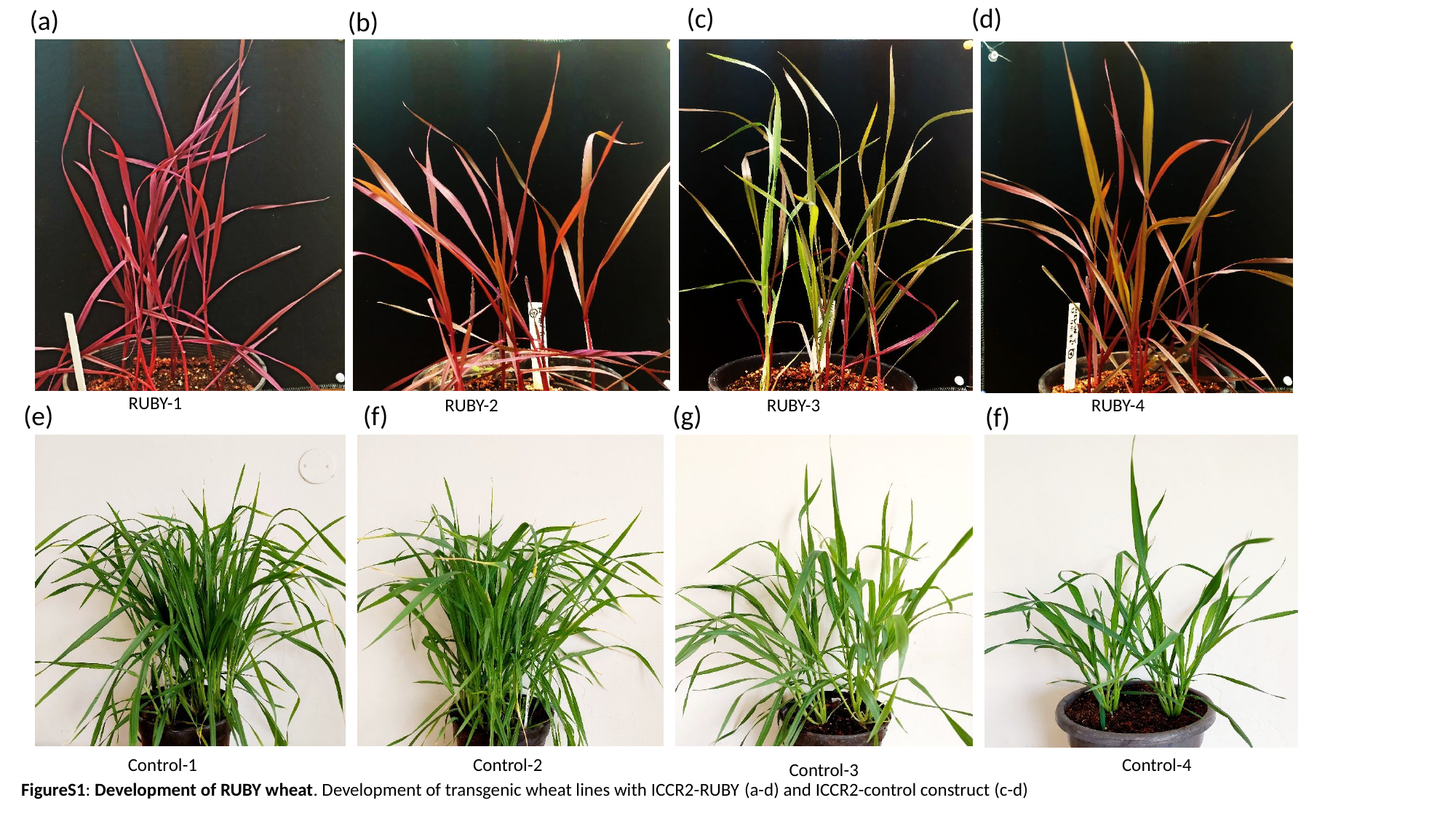

(c)
(d)
(a)
(b)
RUBY-1
RUBY-4
RUBY-2
RUBY-3
(e)
(f)
(g)
(f)
Control-1
Control-2
Control-4
Control-3
 FigureS1: Development of RUBY wheat. Development of transgenic wheat lines with ICCR2-RUBY (a-d) and ICCR2-control construct (c-d)

## Slide 2
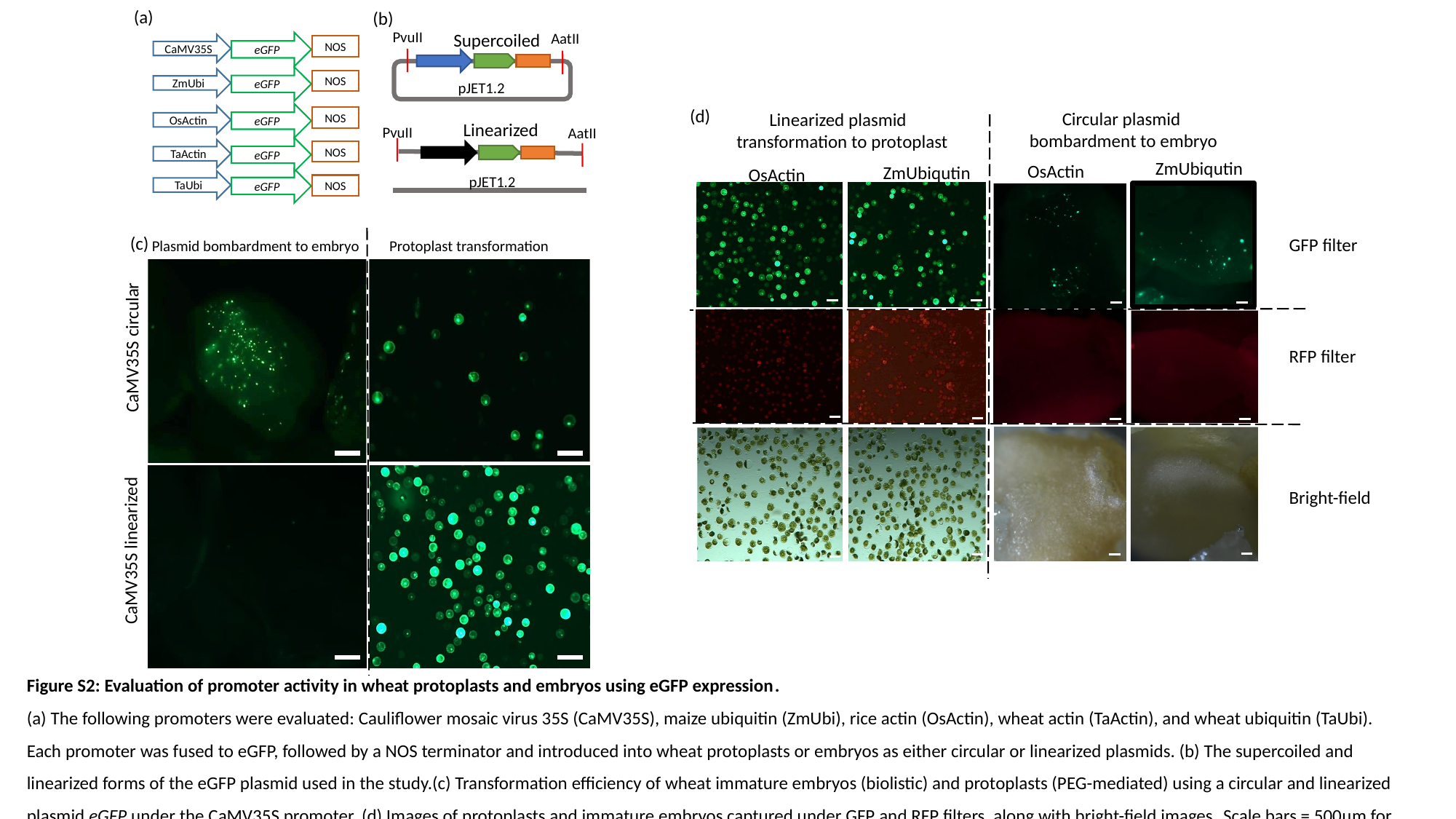

(a)
(b)
PvuII
AatII
Supercoiled
eGFP
CaMV35S
NOS
eGFP
ZmUbi
NOS
eGFP
OsActin
NOS
eGFP
TaActin
NOS
NOS
eGFP
TaUbi
pJET1.2
(d)
Circular plasmid
bombardment to embryo
Linearized plasmid
transformation to protoplast
ZmUbiqutin
OsActin
ZmUbiqutin
OsActin
GFP filter
RFP filter
Bright-field
Linearized
PvuII
AatII
pJET1.2
(c)
 Protoplast transformation
 Plasmid bombardment to embryo
CaMV35S circular
CaMV35S linearized
Figure S2: Evaluation of promoter activity in wheat protoplasts and embryos using eGFP expression.
(a) The following promoters were evaluated: Cauliflower mosaic virus 35S (CaMV35S), maize ubiquitin (ZmUbi), rice actin (OsActin), wheat actin (TaActin), and wheat ubiquitin (TaUbi). Each promoter was fused to eGFP, followed by a NOS terminator and introduced into wheat protoplasts or embryos as either circular or linearized plasmids. (b) The supercoiled and linearized forms of the eGFP plasmid used in the study.(c) Transformation efficiency of wheat immature embryos (biolistic) and protoplasts (PEG-mediated) using a circular and linearized plasmid eGFP under the CaMV35S promoter. (d) Images of protoplasts and immature embryos captured under GFP and RFP filters, along with bright-field images. Scale bars = 500µm for wheat embryos (left) and 100 µm for wheat protoplasts (right)

## Slide 3
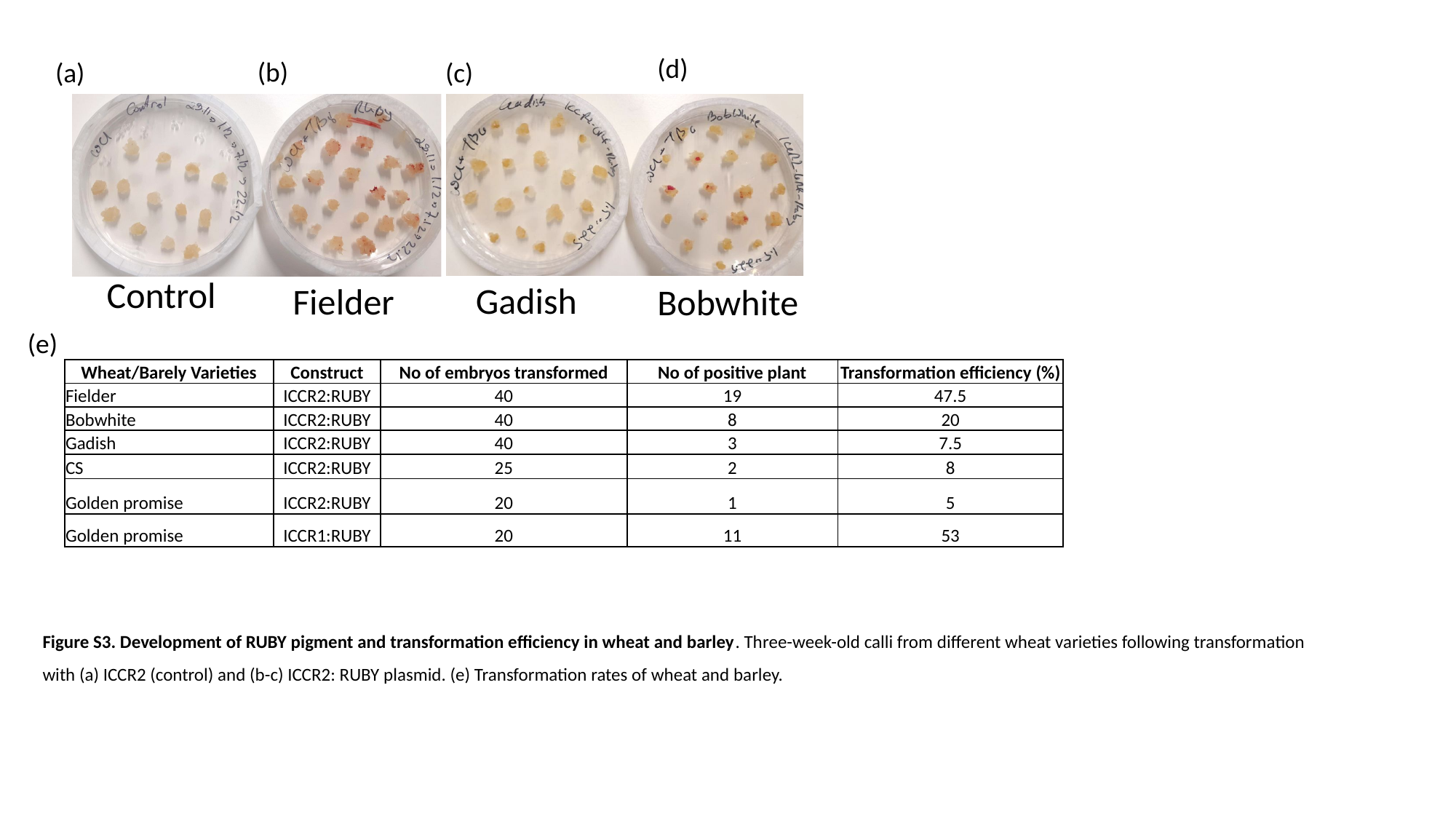

(d)
(b)
(a)
(c)
Control
Gadish
Fielder
Bobwhite
(e)
| Wheat/Barely Varieties | Construct | No of embryos transformed | No of positive plant | Transformation efficiency (%) |
| --- | --- | --- | --- | --- |
| Fielder | ICCR2:RUBY | 40 | 19 | 47.5 |
| Bobwhite | ICCR2:RUBY | 40 | 8 | 20 |
| Gadish | ICCR2:RUBY | 40 | 3 | 7.5 |
| CS | ICCR2:RUBY | 25 | 2 | 8 |
| Golden promise | ICCR2:RUBY | 20 | 1 | 5 |
| Golden promise | ICCR1:RUBY | 20 | 11 | 53 |
Figure S3. Development of RUBY pigment and transformation efficiency in wheat and barley. Three-week-old calli from different wheat varieties following transformation with (a) ICCR2 (control) and (b-c) ICCR2: RUBY plasmid. (e) Transformation rates of wheat and barley.

## Slide 4
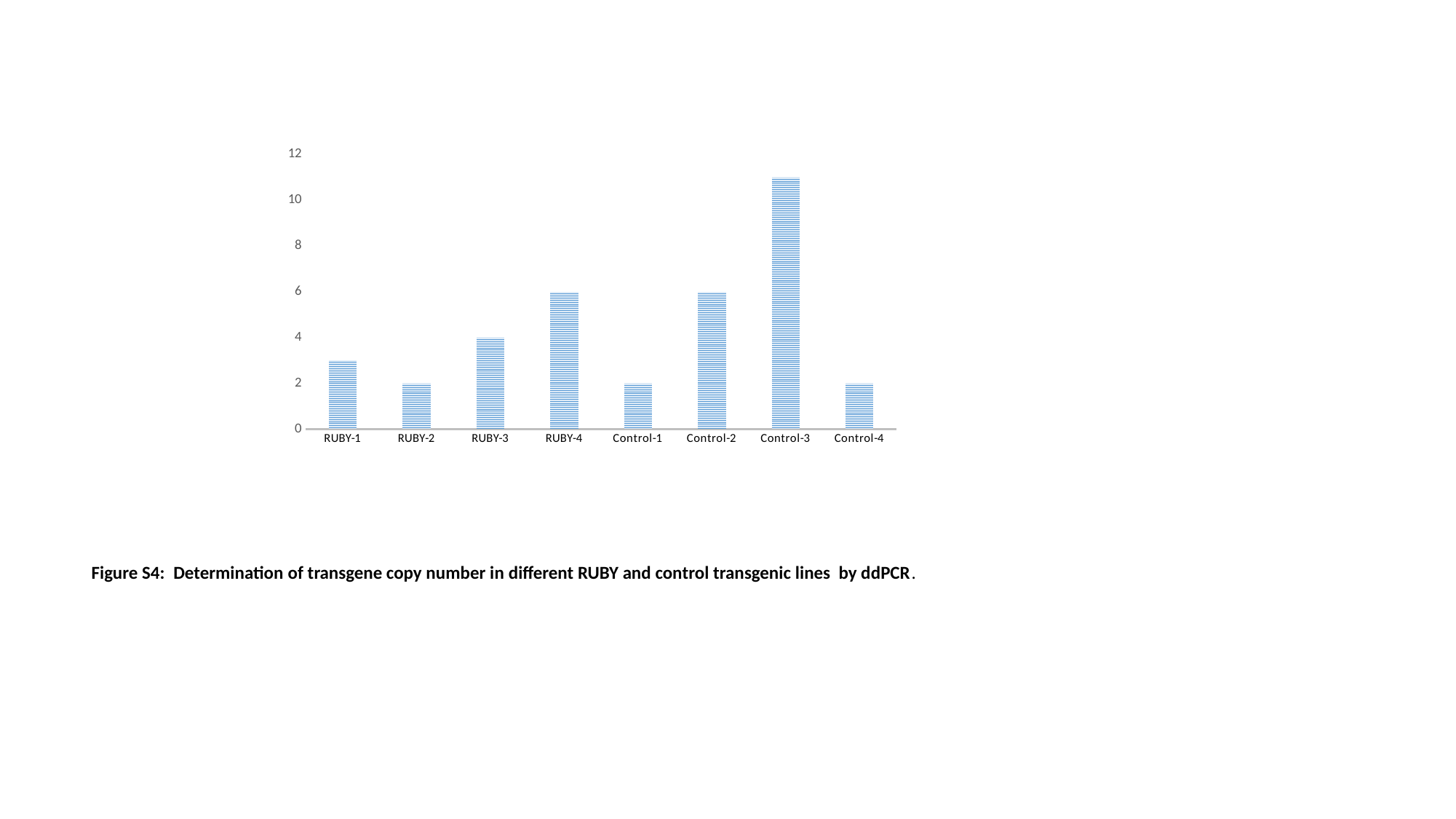

### Chart
| Category | |
|---|---|
| RUBY-1 | 3.0 |
| RUBY-2 | 2.0 |
| RUBY-3 | 4.0 |
| RUBY-4 | 6.0 |
| Control-1 | 2.0 |
| Control-2 | 6.0 |
| Control-3 | 11.0 |
| Control-4 | 2.0 |Figure S4: Determination of transgene copy number in different RUBY and control transgenic lines by ddPCR.

## Slide 5
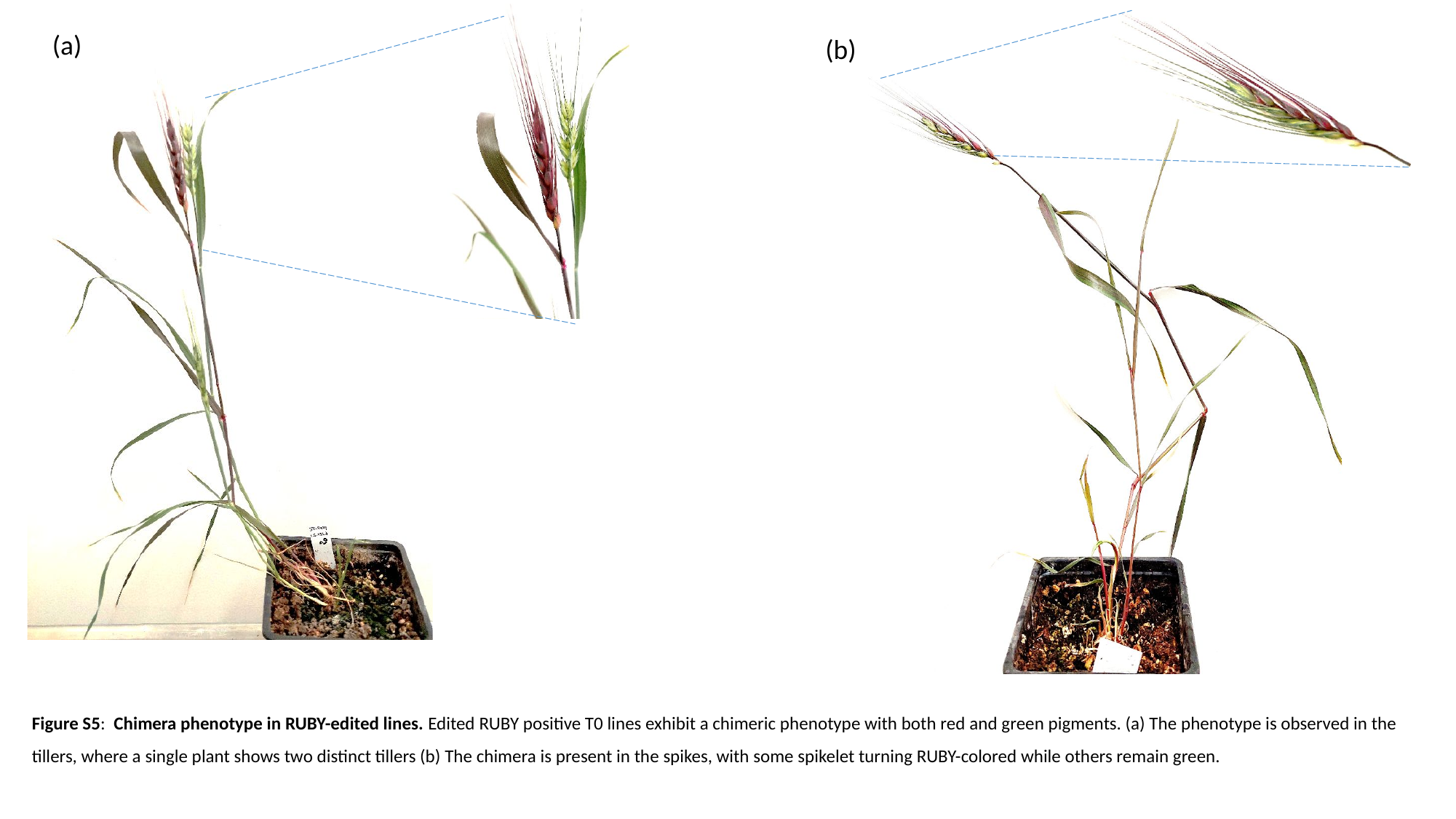

(a)
(b)
(d)
T0-26
T0-22
T0-20
Figure S5: Chimera phenotype in RUBY-edited lines. Edited RUBY positive T0 lines exhibit a chimeric phenotype with both red and green pigments. (a) The phenotype is observed in the tillers, where a single plant shows two distinct tillers (b) The chimera is present in the spikes, with some spikelet turning RUBY-colored while others remain green.

## Slide 6
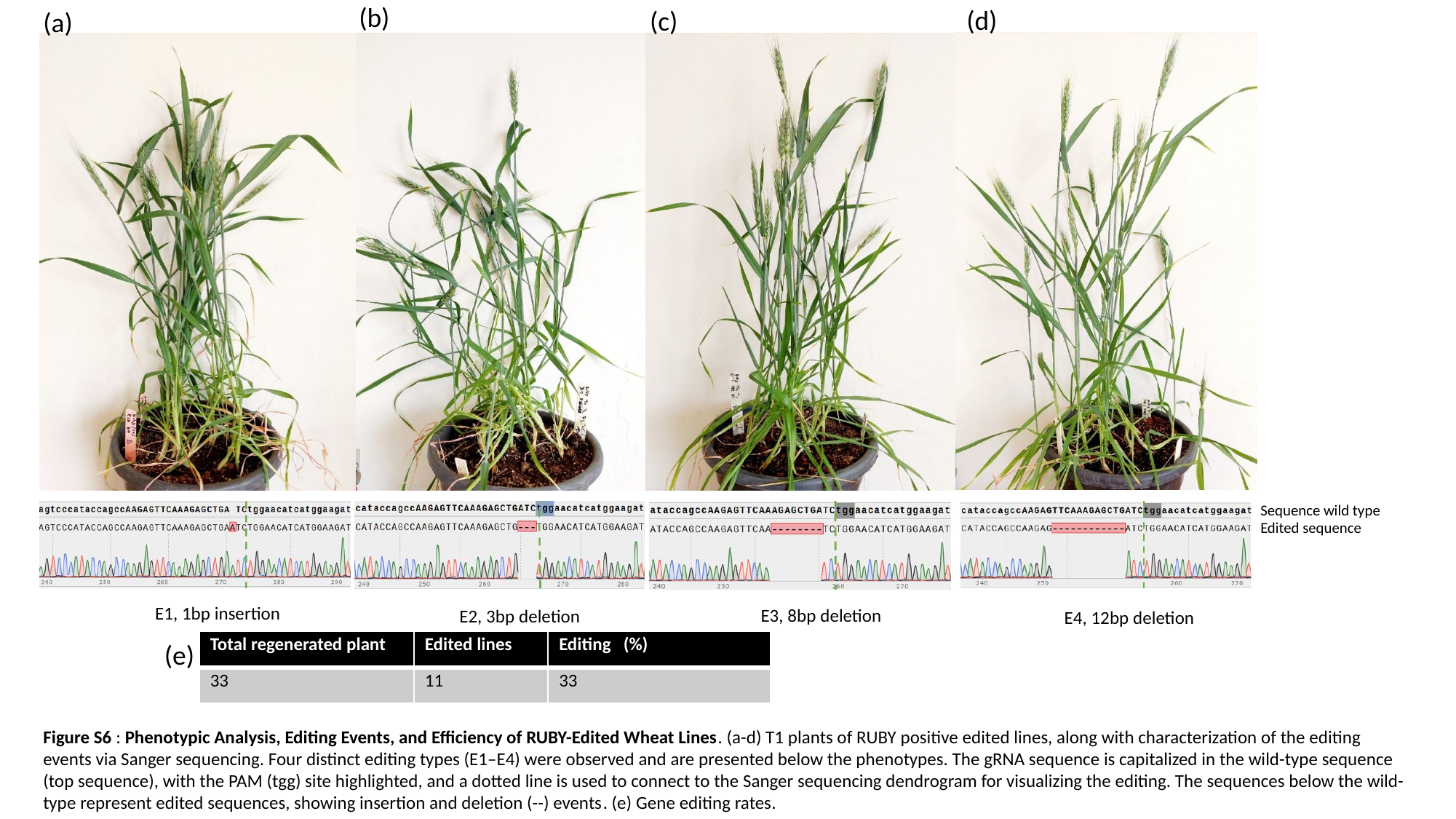

(a)
(b)
(d)
(c)
E1, 1bp insertion
E3, 8bp deletion
E2, 3bp deletion
E4, 12bp deletion
Sequence wild type
Edited sequence
| Total regenerated plant | Edited lines | Editing (%) |
| --- | --- | --- |
| 33 | 11 | 33 |
(e)
Figure S6 : Phenotypic Analysis, Editing Events, and Efficiency of RUBY-Edited Wheat Lines. (a-d) T1 plants of RUBY positive edited lines, along with characterization of the editing events via Sanger sequencing. Four distinct editing types (E1–E4) were observed and are presented below the phenotypes. The gRNA sequence is capitalized in the wild-type sequence (top sequence), with the PAM (tgg) site highlighted, and a dotted line is used to connect to the Sanger sequencing dendrogram for visualizing the editing. The sequences below the wild-type represent edited sequences, showing insertion and deletion (--) events. (e) Gene editing rates.

## Slide 7
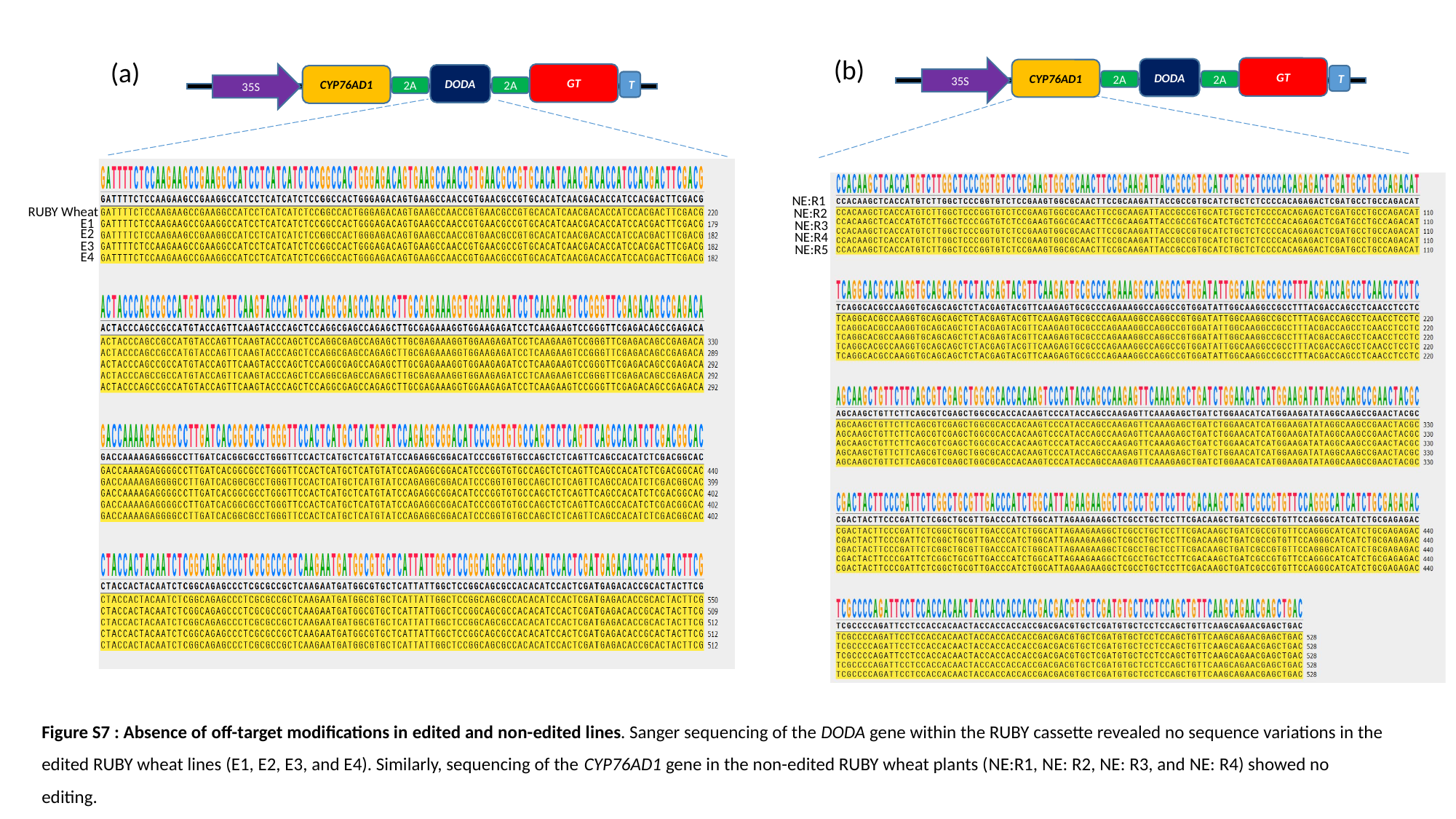

(b)
(a)
35S
GT
DODA
CYP76AD1
T
2A
2A
35S
GT
DODA
CYP76AD1
T
2A
2A
RUBY Wheat
E1
E2
E3
E4
NE:R1
NE:R2
NE:R3
NE:R4
NE:R5
Figure S7 : Absence of off-target modifications in edited and non-edited lines. Sanger sequencing of the DODA gene within the RUBY cassette revealed no sequence variations in the edited RUBY wheat lines (E1, E2, E3, and E4). Similarly, sequencing of the CYP76AD1 gene in the non-edited RUBY wheat plants (NE:R1, NE: R2, NE: R3, and NE: R4) showed no editing.

## Slide 8
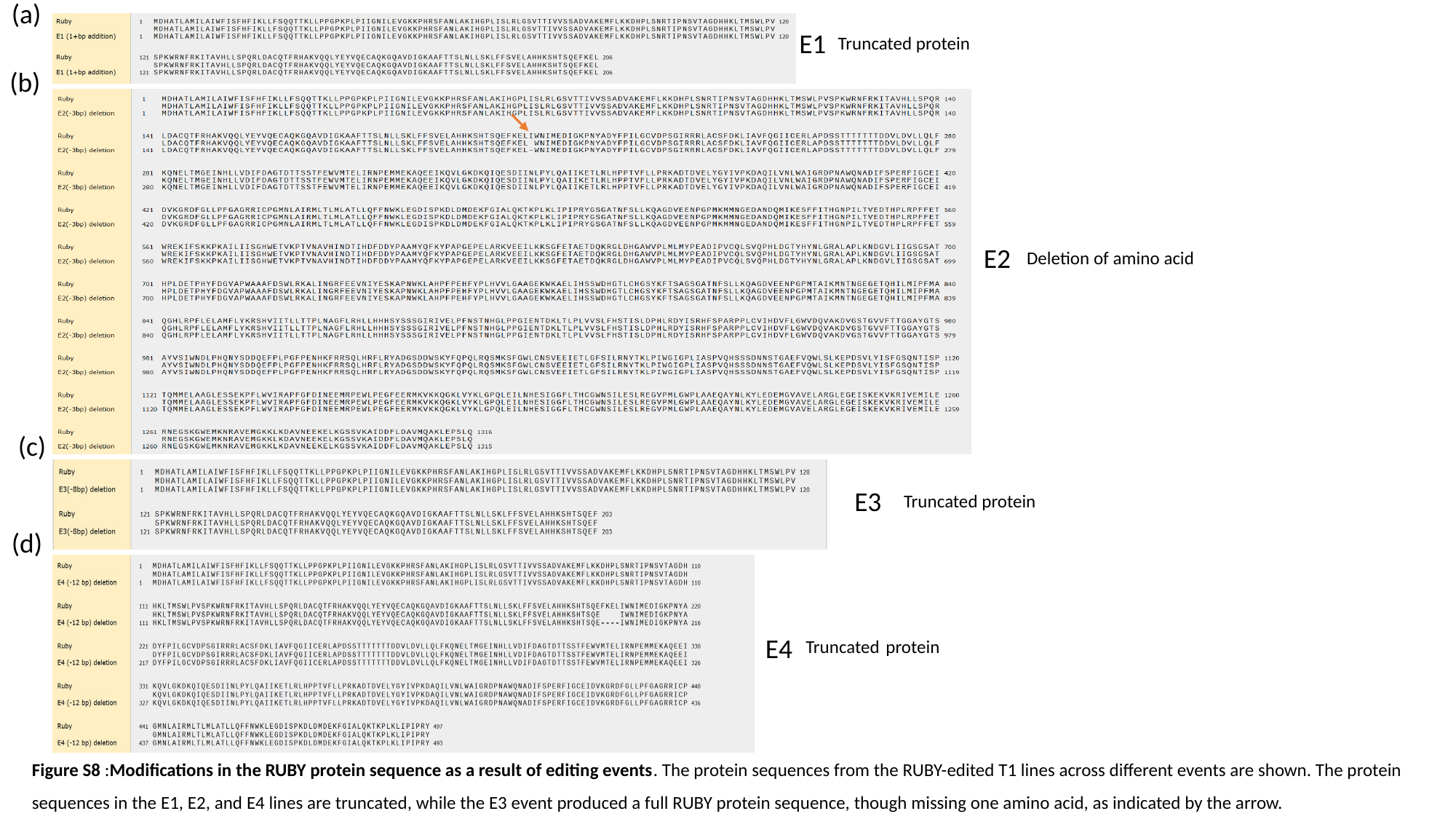

(a)
E1
Truncated protein
(b)
E2
Deletion of amino acid
(c)
E3
Truncated protein
(d)
Truncated protein
E4
Figure S8 :Modifications in the RUBY protein sequence as a result of editing events. The protein sequences from the RUBY-edited T1 lines across different events are shown. The protein sequences in the E1, E2, and E4 lines are truncated, while the E3 event produced a full RUBY protein sequence, though missing one amino acid, as indicated by the arrow.

## Slide 9
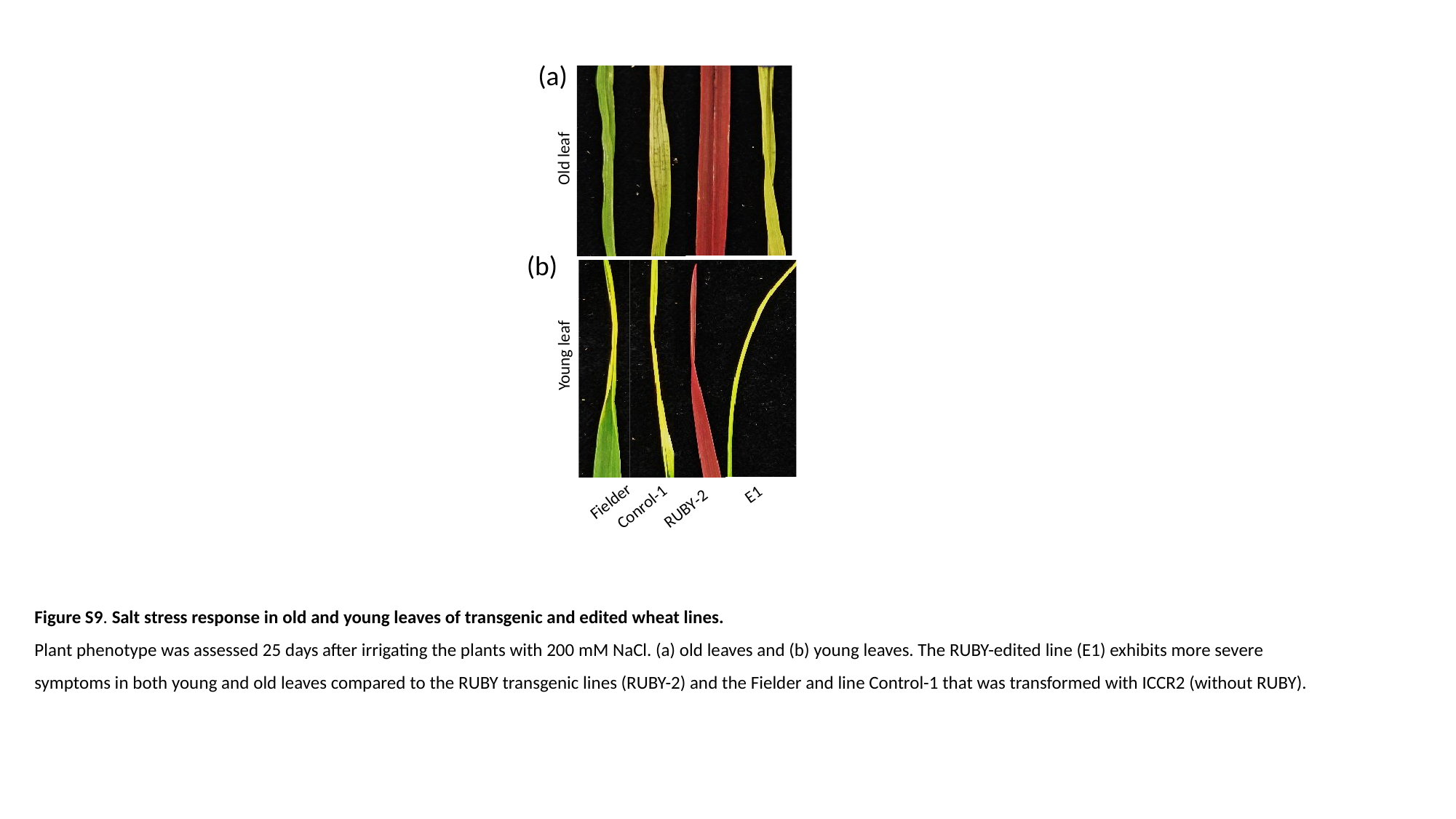

(a)
Old leaf
(b)
Young leaf
RUBY-2
Conrol-1
 E1
Fielder
Figure S9. Salt stress response in old and young leaves of transgenic and edited wheat lines.
Plant phenotype was assessed 25 days after irrigating the plants with 200 mM NaCl. (a) old leaves and (b) young leaves. The RUBY-edited line (E1) exhibits more severe symptoms in both young and old leaves compared to the RUBY transgenic lines (RUBY-2) and the Fielder and line Control-1 that was transformed with ICCR2 (without RUBY).

## Slide 10
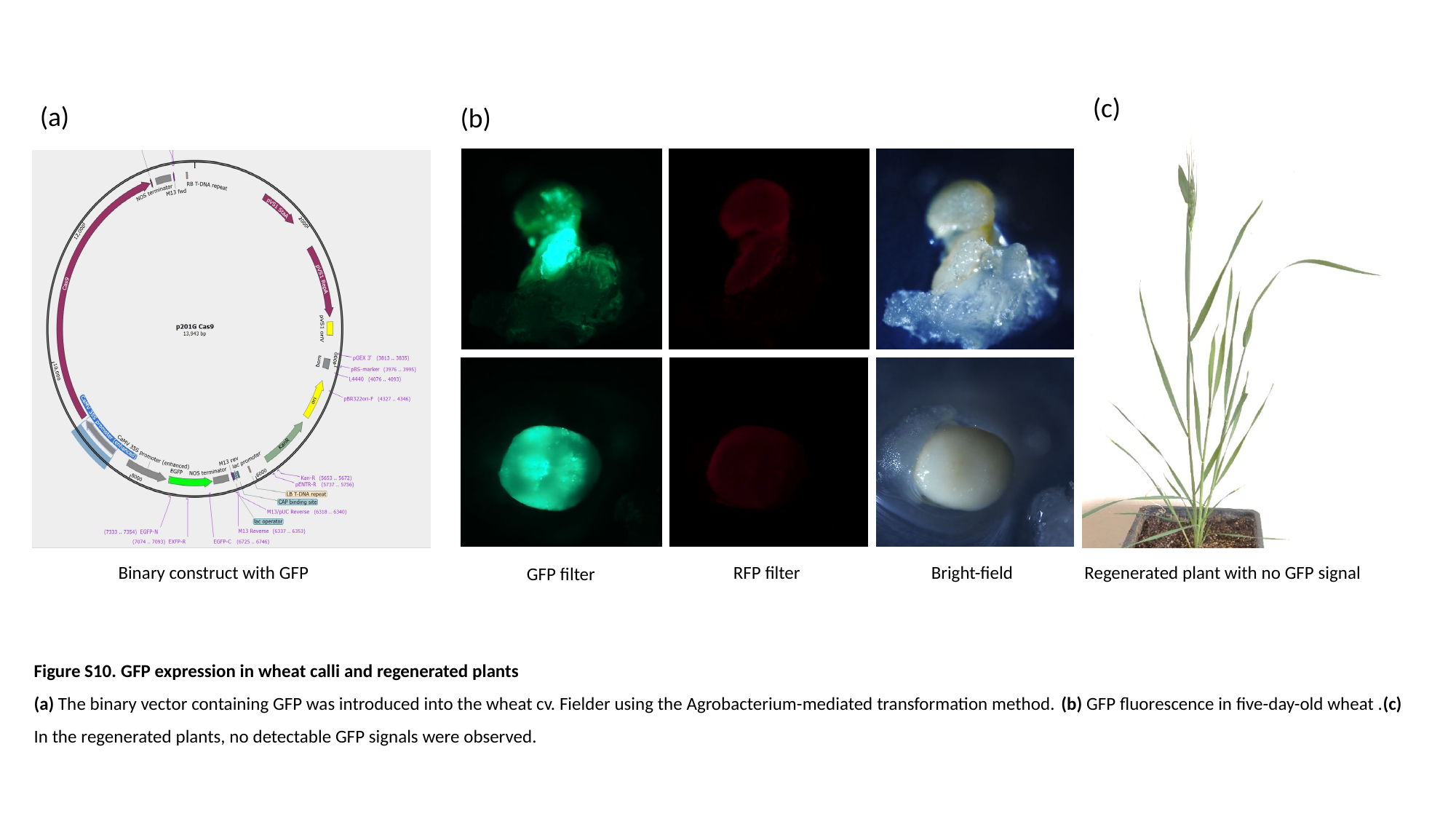

(c)
(a)
(b)
Binary construct with GFP
RFP filter
Regenerated plant with no GFP signal
Bright-field
GFP filter
Figure S10. GFP expression in wheat calli and regenerated plants
(a) The binary vector containing GFP was introduced into the wheat cv. Fielder using the Agrobacterium-mediated transformation method. (b) GFP fluorescence in five-day-old wheat .(c) In the regenerated plants, no detectable GFP signals were observed.

## Slide 11
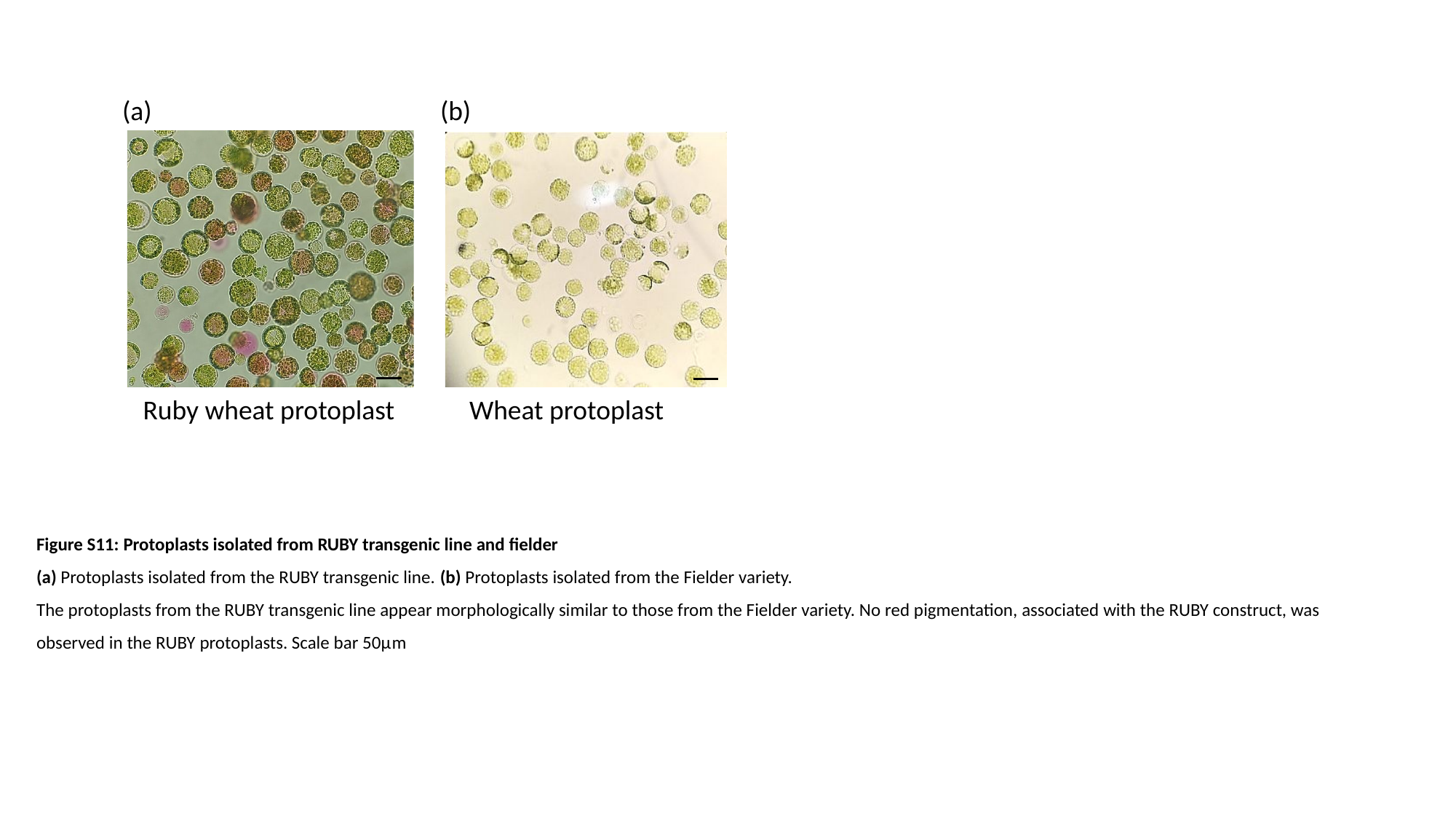

(a)
(b)
Ruby wheat protoplast
Wheat protoplast
Figure S11: Protoplasts isolated from RUBY transgenic line and fielder
(a) Protoplasts isolated from the RUBY transgenic line. (b) Protoplasts isolated from the Fielder variety.
The protoplasts from the RUBY transgenic line appear morphologically similar to those from the Fielder variety. No red pigmentation, associated with the RUBY construct, was observed in the RUBY protoplasts. Scale bar 50µm

## Slide 12
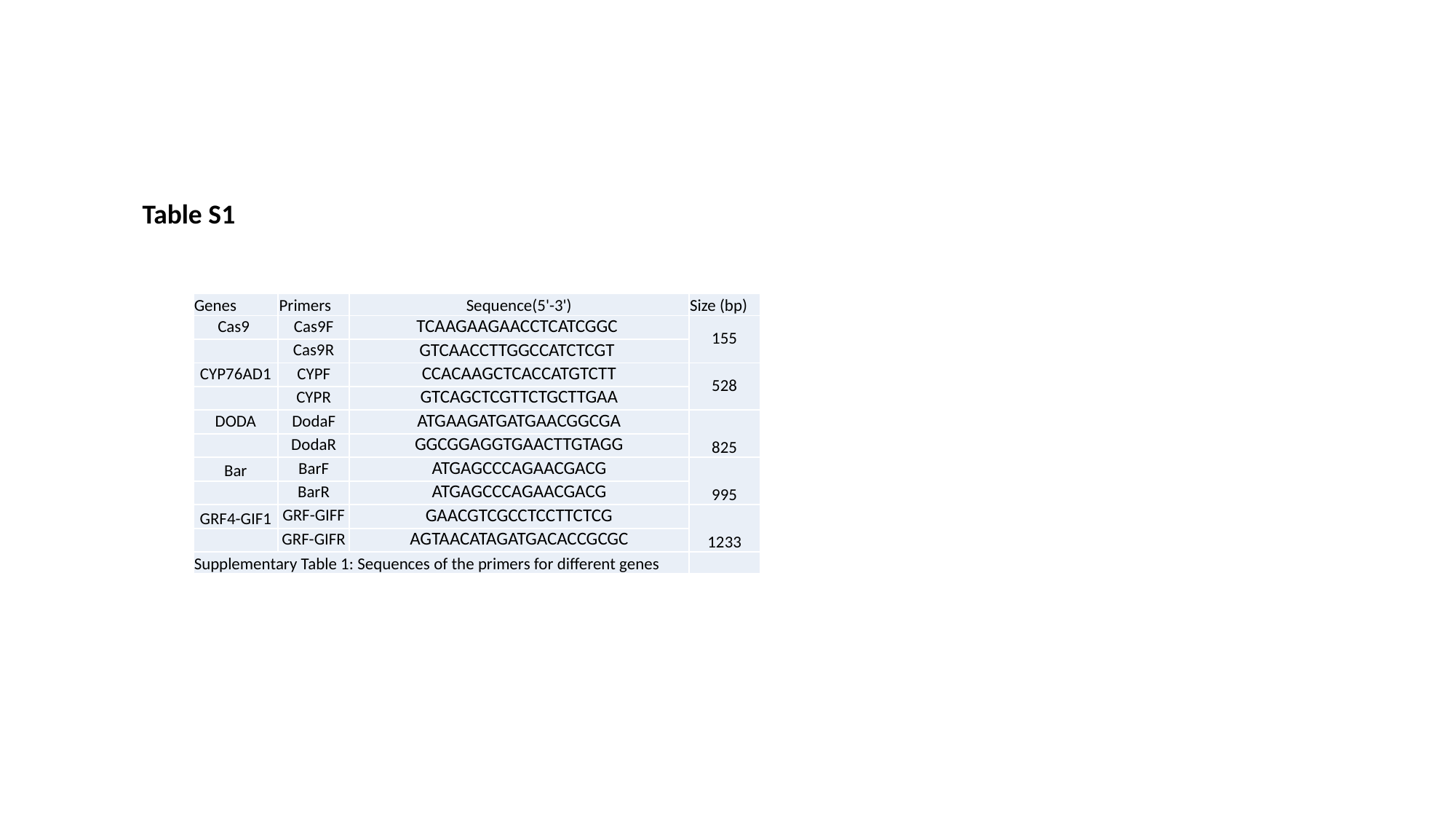

Table S1
| Genes | Primers | Sequence(5'-3') | Size (bp) |
| --- | --- | --- | --- |
| Cas9 | Cas9F | TCAAGAAGAACCTCATCGGC | 155 |
| | Cas9R | GTCAACCTTGGCCATCTCGT | |
| CYP76AD1 | CYPF | CCACAAGCTCACCATGTCTT | 528 |
| | CYPR | GTCAGCTCGTTCTGCTTGAA | |
| DODA | DodaF | ATGAAGATGATGAACGGCGA | 825 |
| | DodaR | GGCGGAGGTGAACTTGTAGG | |
| Bar | BarF | ATGAGCCCAGAACGACG | 995 |
| | BarR | ATGAGCCCAGAACGACG | |
| GRF4-GIF1 | GRF-GIFF | GAACGTCGCCTCCTTCTCG | 1233 |
| | GRF-GIFR | AGTAACATAGATGACACCGCGC | |
| Supplementary Table 1: Sequences of the primers for different genes | | | |
